# Effect of a Nursing Process Training Program on Nurses’ Knowledge and Skills in Primary Healthcare in Albania: A Quasi-Experimental Study

**DOI:** 10.3390/healthcare14132013

**Published:** 2026-07-06

**Authors:** Sonila Qirko, Florin Leasu, Maria Elena Cocuz, Vasilika Prifti, Emirjona Kiçaj, Rudina Çerçizaj, Liliana Marcela Rogozea

**Affiliations:** 1 Faculty of Medicine, Transilvania University of Brasov, 500036 Brasov, Romania; florinleasu@yahoo.com (F.L.); maria.cocuz@unitbv.ro (M.E.C.); vasilika.prifti@unitbv.ro (V.P.); emirjona.kicaj@unitbv.ro (E.K.); rudina.cercizaj@unitbv.ro (R.Ç.); r_liliana@unitbv.ro (L.M.R.); 2 Faculty of Health, University of Vlora, 9401 Vlora, Albania

**Keywords:** nursing process, primary health care, education, competency-based training, Albania

## Abstract

**Background:** The nursing process provides a structured framework for delivering safe, holistic, and patient-centered care; however, its implementation in primary healthcare settings, particularly in low-resource systems, remains inconsistent due to limited training and institutional support. **Objectives:** This study aimed to evaluate the effectiveness of a structured educational intervention in improving nurses’ knowledge and practical competencies in applying the nursing process in primary healthcare centers in Vlora, Albania. **Methods:** A quasi-experimental study was conducted with 32 nurses from five public primary healthcare centers. Sixteen nurses received a structured training program consisting of theoretical instruction and case-based practice, while sixteen nurses served as a control group. Pre- and post-intervention assessments were performed using standardized questionnaires and skill evaluation tools, and differences were analyzed using nonparametric statistical tests. **Results:** The results showed clear improvements in the intervention group across all domains, after the training. The reported use of the nursing process increased from 62.5% to 100%, while the use of Gordon’s Functional Health Patterns increased from 6.3% to 93.7%. The use of NANDA nursing diagnosis increased from 62.5% to 100%. The proportion of nurses reporting written nursing care plans increased from 62.5% to 93.7%, and the implementation and evaluation of care plans increased from 62.5% to 100%. The control group showed no comparable progress. Nurses who participated in the training also reported increased confidence and consistency in applying the nursing process in daily practice. **Conclusions:** These findings suggest that structured, competency-based training programs may improve immediate nurses’ theoretical knowledge and practical skills. Such training may contribute to improving the quality of nursing care, but further studies and longer follow-up and patient-related results are needed.

## 1. Introduction

Nursing practice continues to evolve in response to changing population health needs, technological advancements, and increasing expectations for high-quality and patient-centered care. Within this context, the nursing process (NP) remains a fundamental framework that supports structured, individualized, and evidence-based care. It consists of five key steps—assessment, diagnosis, planning, implementation, and evaluation—which guide clinical decision-making and ensure continuity and quality of care [1].

The nursing process is especially important in primary healthcare because nurses are often involved in assessment, health education, follow-up, chronic disease management, and continuity of care [2].

Despite its recognized importance, the implementation of the nursing process in everyday clinical practice remains inconsistent, particularly in primary healthcare settings and in low-resource systems. In many cases, care delivery is still based on routine practices rather than structured approaches, which may lead to fragmented care, reduced efficiency, and variability in patient outcomes [3].

In Albania, the healthcare system has undergone important reforms aimed at improving accessibility and quality of services, especially in primary healthcare [4,5]. However, challenges related to workforce capacity, training opportunities, and organizational support continue to affect nursing practice. Nurses frequently report limited exposure to structured methodologies such as the nursing process, as well as insufficient training in applying standardized frameworks in daily care.

Previous studies have highlighted ongoing efforts to strengthen nursing education and professional development in Albania, emphasizing the need to align clinical practice with international standards [6]. At the same time, international evidence suggests that the consistent use of the nursing process improves clinical decision-making, enhances documentation quality, and supports patient-centered care [7]. However, the extent to which these approaches are implemented in primary healthcare settings remains insufficiently explored.

Failure to consistently apply the nursing process may result in gaps in care planning, reduced patient satisfaction, and missed opportunities to improve safety and clinical outcomes [8]. These challenges are further compounded by organizational factors such as high patient workload, limited resources, and staff turnover, which may hinder the effective implementation of structured care models [9,10].

Educational interventions have been identified as an effective strategy to address these gaps, particularly when they combine theoretical knowledge with practical, case-based learning approaches. Such interventions can enhance both knowledge and clinical competencies, while also increasing nurses’ confidence in applying the nursing process in real-world settings [11,12].

However, evidence on the effectiveness of structured training programs in the Albanian primary healthcare context remains limited. In particular, few studies have evaluated whether structural educational interventions can improve nurses’ knowledge and self-reported skills related to the nursing process in Albania’s primary healthcare centers. There is a need for research that evaluates how targeted interventions can improve the practical application of the nursing process and support more consistent, high-quality care delivery.

For this reason, the present study aims to evaluate the effectiveness of a structured educational intervention designed to improve nurses’ knowledge and practical application of the nursing process in primary healthcare centers in Vlora, Albania. The research question guiding was: does a structured nursing process training program improve nurse’s knowledge and self-reported skills in primary healthcare settings in Albania? The study hypothesizes that nurses who received the training would show grater improvement in knowledge and self-reported skills compared to nurses who did not receive the training.

## 2. Materials and Methods

### 2.1. Study Design

This manuscript was prepared in accordance with the Transparent Reporting of Evaluation with Nonrandomized Designs (TREND) statement. The team used a quasi-experimental design with a control group to evaluate the effectiveness of a structured educational intervention on nurses’ knowledge and practical application of the nursing process. Pre- and post-intervention assessments were conducted to measure changes in knowledge and skills.

It was hypothesized that nurses who received the educational intervention would demonstrate improved knowledge and practical skills related to the nursing process compared with the control group.

### 2.2. Setting and Participants

The study was conducted in five public primary healthcare centers (HCs) in the city of Vlora, Albania. Data collection took place between September and November 2024. The pre-intervention assessment was conducted at the beginning of September. The educational intervention was implemented between October and November through a series of training sessions conducted in the participating healthcare centers. The post-intervention evaluation was done in late November.

**Figure 1:**
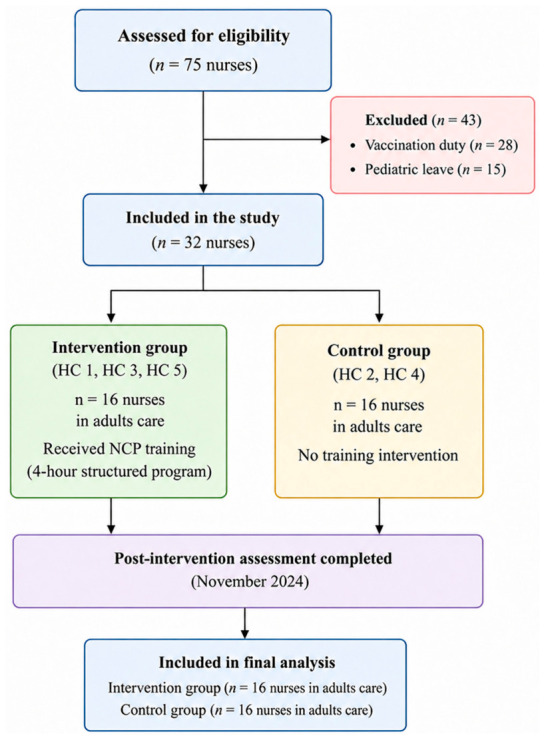
Flowchart of participants’ enrollment, group assignment, and follow-up in the quasi-experimental study.

The number of participants included in the study reflects the actual staffing structure of adult primary healthcare services in the city of Vlora, Albania, which has an estimated population of approximately 144,000 inhabitants. Although five public healthcare centers were included, not all nurses working in these centers were eligible for participation. During the study period, a considerable number of nurses were assigned to vaccination duties or were on pediatric leave, as presented in the participant flow diagram (Figure 1).

The sample size reflected the actual number of eligible and available nurses working in adult primary healthcare services that we included in the study during the data collection period. We did not perform a sample size calculation because our goal was to include all the eligible and available nurses from the participating centers. So, the final sample included a total of 32 nurses who were available during the data collection period.

Participants were not randomly allocated to the intervention and control groups. Group allocation was based on organizational and staffing feasibility during the study period. This approach was chosen to avoid disruption of routine healthcare activities and to reduce the risk of contamination between trained and untrained nurses working within the same centers. For this reason, the groups were assigned by the healthcare center.

The intervention group included all 16 nurses from HC 1, HC 3, and HC 5, who received the training. The control group consisted of all 16 nurses from HC 2 and HC 4, who did not receive the intervention. Nurses on vaccination duty or on pediatric leave during the study period were excluded. However, based on the group separation done by the healthcare center, a possible center effect cannot be excluded. At the end of the study, all the participants in both groups completed the questionnaires pre- and post-intervention, and nobody left the study. All the questionnaires collected were filled in completely.

### 2.3. Intervention

The educational intervention consisted of a structured training program focused on improving nurses’ knowledge and practical application of the nursing process in primary healthcare settings. Each phase of the nursing process was addressed in separate 4 h training sessions. The nursing process has five phases so the team decided to have 4 h nursing sessions for each phase. The 4 h of training for each phase were divided into 2 h of theoretical instruction and 2 h of case-based practical sessions. The theoretical part included the main steps of the nursing process, starting with the assessment, nursing diagnosis, planning, implementation, and evaluation. The practical part consisted of clinical cases and exercises based on real cases in order to strengthen critical thinking and practical skills.

The training content was developed based on international standards, including NANDA-I classifications and Gordon’s Functional Health Patterns [13]. The intervention was guided by competency-based learning principles and adult learning approaches, emphasizing active participation, clinical reasoning, and practical application of knowledge in real clinical scenarios [14,15]. The educational materials were reviewed by two nurse educators and one clinical supervisor to ensure content validity and relevance to primary healthcare practice. The sessions were delivered using interactive discussions and case-based learning approaches to promote active engagement and practical understanding.

The educational intervention was delivered by four researchers with experience in nursing care and nursing education. The training sessions were conducted in designated educational areas within the participating healthcare settings in small groups in order to facilitate interaction and discussion among nurses.

No adverse events were reported during the study period.

### 2.4. Data Collection and Instruments

Data were collected using a structured, self-administered questionnaire applied before and after the intervention. The instrument was adapted from a previously validated tool [3] and included five sections with 54 items in total, including:

- Sociodemographic characteristics;
- Nursing process implementation;
- Organizational and nurse-related factors;
- Knowledge assessment;
- Skill assessment.

The nursing process implementation section assessed the use of the nursing process in daily practice, assessment approaches, NANDA nursing diagnosis, care planning, implementation, and evaluation. The knowledge section included multiple-choice questions related to the stages of the nursing process, Gordon’s Functional Health patterns, nursing diagnosis, planning, implementation and evaluation. The skills sections assessed self-reported nursing skills using a 5-point Likert scale (1 = not at all, 5 = very much).

The instrument was reviewed and adapted for the Albanian primary healthcare context. The adaption process focused on linguistic clarity, cultural appropriateness, and relevance to adult primary healthcare nursing practice. The wording of selected items was adjusted to reflect the terminology and organization of public primary healthcare services in Albania.

Due to the nature of the educational intervention, full blinding was not easy to perform. But during the pre-intervention assessment, nurses were not given information about the group they would belong to. Also, the person responsible for collecting the data post-intervention was not informed about the group to which the nurse was assigned. This was done in order to minimize potential assessment bias.

Content validity was assessed by two experts, and the internal consistency of the knowledge and skills sections showed high reliability (Cronbach’s α = 0.95). The questionnaire was pre-tested on a small group of nurses who were not part of the study. They were part of a healthcare center in Fier, which is close to the city of Vlora and the characteristics of the population of adults and healthcare staffing are similar, and after that the necessary adjustments were made before the main data collection.

### 2.5. Variables

The primary independent variable was the educational intervention. Dependent variables included nurses’ knowledge and practical skills related to the nursing process.

The primary outcomes of the study were changes in nurses’ knowledge and practical skills related to the nursing process following the educational intervention.

Additional variables included sociodemographic and professional characteristics (age, gender, marital status, education level, work experience), as well as organizational factors such as workload, workplace environment, and resource availability.

### 2.6. Statistical Analysis

Data were analyzed using IBM SPSS Statistics version 25.0 (IBM Corp., Armonk, NY, USA). The Shapiro–Wilk test was used to assess the normality of continuous variables. The individual nurse was considered the unit of analysis in this study.

Descriptive statistics were used to summarize the data, including means and standard deviations for continuous variables and percentages for categorical variables.

The Wilcoxon signed-rank test was used to compare pre- and post-intervention scores within groups, while the Mann–Whitney U test was used to compare differences between the intervention and control groups. McNemar’s test was applied for categorical variables.

Effect sizes were calculated to complement statistical significance testing and to quantify the magnitude of the observed differences. For Wilcoxon signed-rank tests and Mann–Whitney U tests, effect size was calculated as r = Z/√N, where Z represents the standardized test statistic and N represents the number of observations included in the analysis. Effect sizes were interpreted as small (r ≈ 0.10), medium (r ≈ 0.30), and large (r ≥ 0.50). A two-tailed *p*-value of <0.05 was considered statistically significant.

A post hoc power analysis was conducted for the within-group pre–post comparisons in the intervention group, using the observed effect size r from the Wilcoxon signed-rank test, with α = 0.05, two-tailed testing, and a sample size of 16 nurses.

### 2.7. Ethical Considerations

The study was conducted in accordance with the principles of the Declaration of Helsinki. Ethical approval was obtained from the Scientific Ethics Committee of the University “Ismail Qemali” (No. 113/1 date 24 April 2024). Permission was also granted by local health authorities in Vlora.

Participation was voluntary, and all participants provided written informed consent. They were informed of their right to withdraw from the study at any time without any consequences.

## 3. Results

### 3.1. Participant Characteristics

A total of 32 nurses participated in the study, equally distributed between the intervention (n = 16) and control group (n = 16). The majority of participants were female, and no statistically significant differences were observed between the groups in terms of sociodemographic and professional characteristics (Table 1). Although small differences were observed in work experience and education level, the groups were considered comparable before the intervention, without statistically significant differences.

### 3.2. Implementation of the Nursing Process

The educational intervention had a significant positive impact on the implementation of the nursing process in daily practice. Before the training, 62.5% of nurses in the intervention group reported using the nursing process, which increased to 100% after the intervention (*p* = 0.031).

The use of standardized approaches also improved substantially. The application of Gordon’s Functional Health Patterns increased from 6.3% to 93.7%, while the use of NANDA classifications for nursing diagnoses rose from 62.5% to 100%. Similarly, the proportion of nurses who reported implementing and evaluating care plans according to expected outcomes increased significantly (Table 2).

### 3.3. Knowledge Outcomes

The training intervention resulted in a significant improvement in nurses’ knowledge of the nursing process. Prior to the intervention, only 56.3% of participants correctly identified data collection (subjective and objective) as the first step of the nursing process; this increased to 100% after training (*p* = 0.015).

Misconceptions observed before training—such as initiating direct interventions or prematurely evaluating patient outcomes—were no longer present after the intervention. Significant improvements were also observed in understanding key concepts, including the purpose of Gordon’s framework, the distinction between nursing and medical diagnoses, and the roles of healthcare professionals within the nursing process (Table 3).

### 3.4. Changes in Knowledge and Skills Scores

Significant improvements were observed across all domains of nursing knowledge and skills following the intervention. Mean scores increased notably in areas such as the application of nursing theories, development of care plans, implementation of interventions, and evaluation of outcomes.

Post-intervention scores indicated near-uniform mastery in several domains, including care planning and intervention implementation (mean = 5.00, SD = 0.00). Substantial improvements were also observed in health and safety practices, patient assessment, and addressing comprehensive patient needs (Table 4). All groups showed large effect sizes, indicating that the observed pre–post improvements were not only statistically significant, but also practically meaningful.

### 3.5. Comparison Between Intervention and Control Groups

Comparative analysis between the intervention and control groups demonstrated significantly higher scores among nurses who received the training across all evaluated domains (*p* < 0.001).

The largest differences were observed in nursing diagnosis, care planning, and evaluation of outcomes, indicating a strong effect of the educational intervention on both knowledge and practical competencies. In contrast, the control group showed consistently lower performance levels across all measures (Table 5). The comparisons between groups were also accompanied by large effect sizes across all domains, supporting the practical relevance of the observed differences between trained and untrained nurses.

Post hoc power ranged from 0.91 to 0.98 across the evaluated items, suggesting adequate power to detect the observed within-group changes. However, because these estimates were based on observed effect sizes and the study included a small sample, they should be interpreted with caution.

## 4. Discussion

The findings of this study indicate that the training program was associated with short-term improvements in knowledge and self-reported skills of nurses related to the nursing process.

However, because the post-test assessment was conducted shortly after the intervention, it is not possible to determine whether these improvements were maintained over time. Therefore, the results should be interpreted as short-term changes in the measured nurse-report outcomes.

One of the main findings was the increased self-report use of the nursing process among nurses in the intervention group after training. This highlights that when nurses are provided with structured education and practical guidance, they are equipped with better understanding and reported application of the main steps of the nursing process. Similar findings have been reported in previous studies, which emphasize that targeted training programs play a key role in promoting the consistent use of the nursing process and improving care quality [11,12].

Another important outcome was the self-reported use of standardized frameworks, such as Gordon’s Functional Health Patterns and NANDA classifications. In the present study, these changes should not be interpreted as evidence of improved care quality, patient-centered care, healthcare delivery, patient outcomes, because these outcomes were not directly assessed. These findings are in line with previous research showing that structured education strengthens the use of standardized nursing languages and contributes to more effective and patient-centered care [7,12,13].

Beyond theoretical knowledge, the intervention was associated with improvement in self-reported practical skills. Nurses demonstrated improved ability to conduct patient assessments, identify accurate nursing diagnoses, develop individualized care plans, and evaluate outcomes collaboratively. These improvements suggest that combining theoretical instruction with case-based learning is particularly effective in bridging the gap between knowledge and practice. Similar conclusions have been reported in studies highlighting the value of experiential and competency-based training approaches in nursing education [11,12,13,14].

The active participation of nurses throughout the training sessions and completion of all intervention activities suggest good implementation fidelity and acceptability of the education program.

The comparison with the control group showed higher post training scores among nurses who received the intervention. However, the lower scores observed in the control group should be interpreted with caution. These differences should not be attributed only to the absence of training, because participants were not randomly allocated, and the groups were separated by healthcare centers. Possible inter-center differences, years of professional experience, workload, staffing patterns, and organizational conditions may have influenced the observed differences between groups. This supports previous research suggesting that continuous professional development is essential for maintaining and improving clinical performance [12,14].

At the same time, the study identified several organizational challenges that may affect the sustainability of these improvements. High patient workload, limited resources, and staff turnover were reported as factors that can hinder the consistent implementation of the nursing process. However, the present study did not measure the long term sustainability of the intervention or its effect on patients’ outcomes.

These findings are consistent with the existing literature, which highlights the impact of systemic and organizational barriers on nursing practice and patient outcomes [9,10].

Although the intervention resulted in significant improvements, some areas, such as goal setting during the planning phase, showed less progress. This suggests that certain components of the nursing process may require more targeted training and repeated practice. Similar challenges have been identified in other studies, where goal formulation is often reported as a complex skill that develops over time and requires continuous reinforcement [15,16,17].

This study has several strengths, including its real-world clinical setting and the use of a structured intervention based on international standards. The inclusion of a control group strengthens the internal validity of the findings and allows for a clearer interpretation of the intervention’s effect. However, some limitations should be acknowledged. The sample size was relatively small and limited to a single geographic area, which may affect the generalizability of the results. No a priori size calculation was performed, because the study included all the eligible and available nurses working in adult primary healthcare services in the participating services during the study period. In addition, participants were not randomly allocated to the intervention and control groups. Group allocation was based on how healthcare centers are allocated for organizational reasons and to reduce the risk of contamination between trained and untrained nurses. However, this approach may have introduced selection bias and the possible center effect.

Furthermore, some basal differences between intervention and control groups were observed, as shown in Table 1. These differences may have influenced the post intervention results, and therefore the findings should not be attributed solely to the educational intervention. In addition, the post-intervention evaluation was conducted shortly after the training, and therefore does not provide information on long-term retention of knowledge and skills.

The instrument we used showed an excellent internal consistency with a Cronbach’s alpha of 0.95, which is a very high value, suggesting possible overlap or redundancy among some items. Therefore, these reliability results should be interpreted with caution. Future studies should further examine the dimensionality and item performance of the questionnaire in the larger sample. In Table 4, several post training scores reached the maximum possible value, with a mean of 5.00 and a SD of 0.00. This suggests a potential ceiling effect meaning that the instrument may have had limited sensitivity to detect further improvements after the intervention. Therefore, although the post training results indicate high self-reported skills, these findings should be interpreted cautiously.

The study did not assess patient outcomes, objective clinical practice, quality of care, patient-centered care, or healthcare delivery. For this reason, the conclusions are limited to nurses’ knowledge and self-reported skills related to the nursing process.

In addition, the post-intervention evaluation was conducted immediately after completion of the educational intervention, so the study could not assess whether the observed improvements in knowledge and self-reported skills were retained over time. Future studies should include follow-up assessments after several weeks or months to evaluate the long term retention of knowledge and skills.

Despite these limitations, the findings may be relevant to similar primary healthcare settings, particularly in resource-limited environments where nurses may have limited access to continuing professional education and structured nursing process training [18].

The findings of this study show that the structured educational intervention was associated with improvements in nurses’ knowledge, self-reported skills, and self-reported implementation of the nursing process. After the training, nurses in the intervention group reported greater use of nursing assessment, nursing diagnosis, care planning, implementation, and evaluation in routine practice. These results suggest that targeted educational programs may support nurses in applying the nursing process more consistently in primary care settings.

However, the present study did not directly assess patient outcomes, quality of care, patient-centered care, or healthcare delivery indicators. Therefore, the interpretation of the findings should remain limited to the measured outcomes: nurses’ knowledge, self-reported skills, and self-reported use of the nursing process.

Future studies with larger sample sizes and longer follow-up periods, random allocation and objective measures of nursing practice are needed to evaluate whether similar training programs are associated with sustained changes in practice- or patient-level outcomes.

## 5. Conclusions

This study found that a structured educational intervention was associated with significant improvements in nurses’ knowledge, self-reported skills, and self-reported implementation of the nursing process. The results indicate that training focused on nursing process steps, NANDA-I nursing diagnoses, and Gordon’s Functional Health Patterns may help nurses apply the nursing process more consistently in daily practice.

Due to the small sample size, nonrandomized design, short-term follow-up, and reliance on self-reported measures, the findings should be interpreted with caution. Further studies with larger samples, longer follow-up periods, and direct assessment of patient outcomes are needed.

## Figures and Tables

**Table 1:** Sociodemographic and professional characteristics of the participants in the intervention and control group.

Variables	Intervention Group n (%)		Control Group n (%)		*p*
Gender					0.15
Female	14	87.5	16	100.0	
Male	2	12.5			
Age group					0.118
<29	3	18.8	1	6.2	
30–39	5	31.2	2	12.5	
40–49	7	43.7	7	43.7	
50–59	1	6.2	6	37.5	
Marital status					0.078
Cohabiting	2	12.5			
Married	8	50.0	12	75.0	
Single	6	37.5	2	12.5	
Divorced			2	12.5	
Health facility ^†^					<0.001
HC 1	7	43.7			
HC 2			9	56.2	
HC 3	6	37.5			
HC 4			7	43.7	
HC 5	3	18.8			
Morning shift	16	100.0	16	100.0	n/a
Work experience					0.067
<5 yr	4	25.0	1	6.2	
5–10 yr	5	31.2	1	6.2	
11–20 yr	4	25.0	6	37.5	
>20 yr	3	18.8	8	50.0	
Education level					0.49
Bachelor	6	37.5	3	18.8	
Master of Science	5	31.2	7	43.7	
Professional Master	5	31.2	6	37.5	

^†^ Health center.

**Table 2:** Nursing process implementation.

Items	Before Training n (%)	After Training n (%)	*p*
The use of the nursing process in daily nursing care	10 (62.5)	16 (100.0)	0.031
If yes, what types of assessments were used?			<0.001
Body system approach	8 (50.0)	1 (6.2)	
Gordon approach	1 (6.3)	15 (93.7)	
Head to toes approach	1 (6.3)	0	
If yes, was NANDA ^†^ used for nursing diagnoses?	10 (62.5)	16 (100.0)	0.031
If yes, is a nursing care plan written with goals, outcomes, and interventions?	10 (62.5)	15 (93.7)	0.125
If yes, is the care plan implemented and evaluated?	10 (62.5)	16 (100.0)	0.031
If yes, is implementation evaluated against expected outcomes?	10 (62.5)	16 (100.0)	0.031

^†^ North American Nursing Diagnosis Association.

**Table 3:** Knowledge assessment.

Items	Before Training	After Training	*p*
First step of the nursing process			0.015
Collecting subjective and objective data	9 (56.3)	16 (100.0)	
Directly intervening the problem	4 (25.0)	0	
Evaluating what has been done for the patient	1 (6.2)	0	
Indicating the activities to be done	2 (12.5)	0	
Primary aim of Gordon’s approach?			<0.001
Focuses on ethical principles	2 (12.5)		
Focuses on patient’s attendant interest	1 (6.2)		
Focuses on patients’ responses towards their illness	2 (12.5)	16 (100.0)	
Focuses on the disease process/medical diagnosis	11 (68.8)		
Which is not a component of the nursing process?			0.007
Assessment	2 (12.5)		
Evidenced based practice	8 (50.0)	16 (100.0)	
Implementation	1 (6.2)		
Planning	5 (31.3)		
How is nursing diagnosis different from medical diagnosis?			<0.001
Both focus on patient’s responses	1 (6.2)		
Both have similar procedures	9 (56.3)		
Nursing Dx. focuses more on diseases than on patient responses	6 (37.5)	1 (6.2)	
Nursing Dx. focuses on the patient’s response		15 (93.7)	
Who is key to the nursing process?			<0.001
All	9 (56.3)		
Nurses		16 (100.0)	
Physician	7 (43.8)		
Activities during planning phase?			0.904
Assigning priorities	2 (12.5)		
Identifying interdependent interventions	1 (6.2)		
Recording the data of the patient	1 (6.2)		
Specifying expected outcomes	1 (6.2)	2 (12.5)	
Specifying goals	11 (68.8)	14 (87.5)	
Role in implementation phase?			<0.001
Implementing the proposed interventions	1 (6.2)	16 (100.0)	
Performing planned interventions, excluding daily living activities	6 (37.5)		
Propose the interventions	7 (43.8)		
Stop the phase if initial interventions fail to resolve the problem	2 (12.5)		

**Table 4:** Nursing knowledge and skills before and after training.

Items	Before Training		After Training		*p*	Effect Size r	Approximate Post Hoc Power
	M	SD	M	SD			
Application of Nursing Theories	3.69	1.40	5.00	0.00	0.005	0.92	0.96
Maintaining Dignity, Privacy, and Confidentiality	4.31	0.70	5.00	0.00	0.005	0.94	0.96
Health and Safety Practices	3.63	0.81	4.75	0.45	0.0003	0.93	0.96
Medication and Therapy Administration	4.44	0.51	4.94	0.25	0.004	1.00	0.98
Addressing Comprehensive Patient Needs	3.06	1.29	4.88	0.34	0.0007	0.90	0.95
Conducting Proper Assessments	3.50	1.55	4.94	0.25	0.006	0.90	0.95
Identifying Accurate Nursing Diagnoses	3.31	1.78	4.38	0.50	0.03	0.82	0.91
Developing Nursing Care Plans	3.50	1.59	5.00	0.00	0.007	0.90	0.95
Effective Implementation of Interventions	3.56	1.36	5.00	0.00	0.004	0.91	0.95
Evaluating Outcomes Collaboratively	3.00	1.15	5.00	0.00	0.0004	0.89	0.95

Note: effect size was calculated as r = Z/√N for the Wilcoxon signed-rank test. Values of approximately 0.10, 0.30 and ≥0.50 indicate small, moderate and large effects, respectively.

**Table 5:** Knowledge and skills scores: intervention vs. control group.

Items	Intervention Group		Control Group		*p*	Effect Size r
	M	SD	M	SD		
Application of Nursing Theories	5.00	0.00	3.06	1.48	*p* < 0.001	0.81
Maintaining Dignity, Privacy, and Confidentiality	5.00	0.00	4.44	0.51	*p* < 0.001	0.63
Health and Safety Practices	4.75	0.45	3.19	1.38	*p* < 0.001	0.69
Medication and Therapy Administration	4.94	0.25	4.25	0.45	*p* < 0.001	0.71
Addressing Comprehensive Patient Needs	4.88	0.34	2.38	1.46	*p* < 0.001	0.86
Conducting Proper Assessments	4.94	0.25	3.06	1.48	*p* < 0.001	0.72
Identifying Accurate Nursing Diagnoses	4.38	0.50	2.25	1.53	*p* < 0.001	0.76
Developing Nursing Care Plans	5.00	0.00	2.75	1.24	*p* < 0.001	0.91
Effective Implementation of Interventions	5.00	0.00	2.75	1.48	*p* < 0.001	0.91
Evaluating Outcomes Collaboratively	5.00	0.00	2.63	1.63	*p* < 0.001	0.86

Note: effect size was calculated as r = Z/√N for the Mann–Whitney U test. Values of approximately 0.10, 0.30 and ≥0.50 indicate small, moderate and large effects, respectively.

## Data Availability

The data presented in this study are available on request from the corresponding author due to privacy and ethical restrictions. Given the relatively small sample size and the inclusion of the participants from the specific primary healthcare centers, public sharing of the data could increase the risk of indirect participants’ information.

## Abbreviations

The following abbreviations are used in this manuscript:

HC	Health center
NANDA	North American Nursing Diagnosis Association
NANDA I	NANDA International
NP	Nursing Process
PHC	Primary Health Care
SD	Standard Deviation
WHO	World Health Organization
